# Clotrimazole for Vulvovaginal Candidosis: More Than 45 Years of Clinical Experience

**DOI:** 10.3390/ph13100274

**Published:** 2020-09-25

**Authors:** Werner Mendling, Maged Atef El Shazly, Lei Zhang

**Affiliations:** 1 German Center for Infections in Obstetrics and Gynaecology, Heusnerstrasse 40, D-42283 Wuppertal, Germany; w.mendling@t-online.de; 2 Bayer Consumer Care AG, Peter Merian-Strasse 84, CH-4002 Basel, Switzerland; maged.el-shazly@bayer.com

**Keywords:** canesten, clotrimazole, vulvovaginal, vaginitis, mycosis, candidosis, yeast infection, candida, candida albicans, vaginal health

## Abstract

Vulvovaginal candidosis is a common disease, and various treatment strategies have emerged over the last few decades. Clotrimazole belongs to the drugs of choice for the treatment of vulvovaginal candidosis. Although available for almost 50 years, systematic reviews on the usefulness of topical clotrimazole across disease severity and populations affected are scarce. Thus, we conducted a systematic literature search in the PubMed and Embase databases to summarize the effectiveness and safety of topical clotrimazole in the treatment of uncomplicated (acute) and complicated vulvovaginal candidosis. In total, 37 randomized controlled studies in women suffering from vaginal yeast infections qualified for inclusion in our review. In women with uncomplicated vulvovaginal candidosis, single intravaginal doses of clotrimazole 500 mg vaginal tablets provided high cure rates and were as effective as oral azoles. A single dose of clotrimazole 500 mg was equipotent to multiple doses of lower dose strengths. Prolonged treatment regimens proved to be effective in severe and recurrent cases as well as in symptomatic pregnant women. It is therefore expected that in the general population, clotrimazole will continue to be widely used in the field of vaginal health in the upcoming years; more so as clotrimazole resistance in vaginal candidosis is rare.

## 1. Introduction

Clotrimazole is an imidazole antimycotic agent that was discovered in the 1960s. It has a specific chemical structure consisting of four aromatic rings, out of which one represents an imidazole ring [1]. Clotrimazole has a broad antimicrobial activity against *Candida albicans* and other fungal species. Like other azole-type antifungal drugs, the antimycotic properties are mediated by an interaction with ergosterol synthesis (via inhibition of the fungal cytochrome 14α-demethylase enzyme) eventually resulting in increased fungal cell wall leakiness with disruption of the structure and function of the cell wall [2]. Topical clotrimazole is widely used for the treatment of tinea pedis (athlete’s foot), cutaneous mycoses, and oropharyngeal candidosis [1,3]. It also belongs to the drugs of choice for the topical treatment of vulvovaginal candidosis and *Candida* balanitis [4,5]. 

Clotrimazole was first registered as Canesten^®^ in Germany more than 45 years ago (in 1973) [6]. The initial formulation for local treatment of vulvovaginal candidosis was the vaginal tablet [7] followed by internal vaginal cream, external cream and soft ovule (soft capsule). Additional formulations are marketed under other trade names. Drug combinations (e.g., clotrimazole plus fluconazole) are also available nowadays. Clotrimazole monopreparations for the management of vulvovaginal candidosis are available over the counter in most countries and cover a dose range from 100 to 500 mg (solid systems). Comparable local clotrimazole exposure can be achieved by administration of semi-solid systems (e.g., creams containing clotrimazole 1%, 2% or 10%) to the vagina and vulva [1]. While many preparations are available as generics, Canesten^®^ is still the market leader in this field [8]. 

Clotrimazole has a poor oral bioavailability. When administered intravaginally, approximately 3% of the dose is systemically available [9]. The latter explains the favorable systemic tolerability of clotrimazole following vaginal application.

Approximately 70–75% of childbearing aged women experience symptomatic vulvovaginal candidosis at least once during their life and 40–50% will suffer from repeated episodes during their lifetime. About 5–8% of adult women may experience recurrent vulvovaginal candidosis (i.e., ≥4 episodes per year) [10,11]. *Candida albicans* is the most common pathogen, but other non-albicans *Candida* species may also be causative microorganisms, particularly in association with disease recurrence [8,11]. The treatment of vulvovaginal candidosis depends on whether the infection is uncomplicated or complicated. Complicated cases comprise recurrent, severe and non-albicans vulvovaginal candidosis as well as vulvovaginal yeast infections during pregnancy and in subjects with immunocompromised conditions [4]. According to current treatment guidelines, topical clotrimazole may play a role in the treatment of both uncomplicated and complicated cases [4,12,13,14]. Despite the fact that in certain patient subpopulations resistance to clotrimazole has been reported [1,15], clotrimazole resistance in vaginal candidosis is rare and susceptibility testing is usually not recommended [12,16]. It is therefore expected that in the general population, clotrimazole will continue to be widely used in the field of female intimate health in the upcoming years. 

This article presents the results of a systematic literature search on the effectiveness and safety of topical clotrimazole when used for the treatment of uncomplicated (acute) and complicated vulvovaginal candidosis. In addition, the scientific evidence for its topical use in men with *Candida* balanitis will also be explored.

## 2. Methods

A systematic literature search was performed in the PubMed and Embase databases. The following various combinations of terms were queried: clotrimazole, Canesten, vulvovaginal, vaginal, vaginitis, mycosis, candidiasis, candidosis, yeast infection, balanitis, study. Moreover, references quoted in identified publications were tracked. Articles for this review were restricted to prospective randomized controlled clinical trials in which mycological cure was part of the efficacy endpoints. Active-controlled trials in uncomplicated vulvovaginal candidosis must have included an arm studying an oral imidazole/triazole antifungal agent to judge the relative effectiveness of intra-vaginal clotrimazole in comparison with oral treatment. This approach was in accordance with a previous systematic review on anti-fungal agents for uncomplicated vulvovaginal candidosis [17]. There were no date or size restrictions. In principle, only English written articles were considered. However, since the early studies were frequently conducted in Germany, German written papers were also included in this review. 

## 3. Results

In total, the search identified 273 publications. Of these, 37 articles were included to assess the effectiveness and safety of topical clotrimazole in the treatment of uncomplicated (acute) and complicated vulvovaginal candidosis. Two additional studies conducted in men with *Candida* balanitis also qualified for inclusion. Most common reasons for article rejection were missing randomization or control group, comparator other than oral imidazole/triazole antifungal agents, use of clotrimazole combination products, non-reporting of mycological cure rate, and inclusion of largely mixed populations (e.g., subjects with uncomplicated and complicated disease or pregnant and non-pregnant women); or the publication was out of scope for this review (e.g., experimental or mechanistic studies).

### 3.1. Clotrimazole in Uncomplicated (Acute) Vulvovaginal Candidosis

Approximately 80–90% of symptomatic vulvovaginal candidosis cases are uncomplicated in nature [4]. Among them, about 75–90% are caused by overgrowth of *Candida albicans* [14,18]. The symptoms are of mild to moderate severity and occur in otherwise healthy, non-pregnant women with a frequency of less than four episodes per year [10]. The condition is uncommon in postmenopausal women without hormonal replacement therapy and in premenarchal girls because in these populations, the vagina is infrequently colonized by *Candida* species [12,14,19,20]. In many women of childbearing age, vaginal *Candida* colonization is asymptomatic and needs no treatment [14]. Therefore, the diagnosis of vulvovaginal candidosis necessitates both the presence of symptoms (e.g., itching, burning, inflammation, or dysuria) and proof of *Candida* in the vagina/vulvar region [11]. 

In our systematic review, 27 articles were included that studied intravaginal clotrimazole in adolescents (usually ≥16 years) and adults with uncomplicated (acute) symptomatic vulvovaginal candidosis. Clotrimazole was compared with placebo in three trials and versus an oral imidazole/triazole antifungal agent in 15 studies (Table 1). Ten trials compared different formulations or treatment regimens of clotrimazole (Table 2).

Generally, single doses of clotrimazole 500 mg vaginal tablet provided mycological cure rates of 70–95% at 1 or 2 weeks following treatment. In studies reporting longer-term cure rates (i.e., after approximately 4 weeks), mycological cure rates ranged roughly between 60% and 90%. Once-daily doses of clotrimazole 200 and 100 mg vaginal tablets for 3 and 6–7 days, respectively, provided similar results. The single use of clotrimazole 10% internal cream resulted in a 1-week mycological cure rate of 85–91%. Combined treatment of clotrimazole 500 mg vaginal tablet and clotrimazole 1% external cream led to mycological cure in 80–95% of subjects within 2 weeks after treatment, whereas the combined application of clotrimazole 10% internal cream and 2% external cream yielded a 2-week cure rate of 74% in one study [21]. 

Topical imidazole was more effective than placebo in the treatment of uncomplicated (acute) symptomatic vulvovaginal candidosis. In most head-to-head comparisons, intravaginal clotrimazole showed a comparable effectiveness to oral imidazole/triazole antifungal agents. Specifically, in five trials, single-dose therapies with topical clotrimazole and oral fluconazole were compared. There, clotrimazole 500 mg vaginal tablet provided mycological cure rates of 75–95% at 1 or 2 weeks after therapy compared to 76–87% with oral fluconazole 150 mg. When intravaginal clotrimazole formulations or treatments were compared versus each other, it turned out that a single dose of clotrimazole 500 mg was as effective as multiple doses of lower dose strengths (e.g., 200 mg for three days). Similarly, the single use of clotrimazole 10% internal cream or clotrimazole 500 mg ovule (soft capsule) yielded mycological cure rates that were comparable to those following a single dose of clotrimazole 500 mg vaginal tablet. In cases of vulvar involvement, the combined use of intravaginal clotrimazole and external clotrimazole cream provided more favorable treatment results than intravaginal clotrimazole alone [22]. In all studies, topical clotrimazole was well tolerated. 

Previous systematic reviews or meta-analyses reported similar findings. Watson et al. [17] included 17 studies in a meta-analysis to compare the effectiveness of oral and intravaginal therapies for uncomplicated vulvovaginal candidosis. There were no statistically significant differences between oral and intravaginal treatments in terms of mycological and clinical cure rates. A subsequent Cochrane review on the same subject came to identical conclusions [23]. Two systematic reviews, including more than 20 studies, inferred that intravaginal and oral imidazoles appear to be equally effective in treating uncomplicated vulvovaginal candidosis, and that single-dose regimens may provide similar results as multiple-dose regimens [24,25].

### 3.2. Clotrimazole in Complicated Vulvovaginal Candidosis

#### 3.2.1. Vaginal Yeast Infection during Pregnancy

During pregnancy, up to 50% of women experience vulvovaginal candidosis [51]. In addition, in pregnant women, recurrent vaginal yeast infections and insufficient therapeutic responses are more frequently recorded than in non-pregnant women [10,52]. An increased content of glycogen and lower pH value in the vagina as well as an intensified binding of yeast cells to the vaginal mucosa have been made accountable for this observation [10,51]. During pregnancy, the therapy of symptomatic vaginal yeast infections should be intense, but restricted to topical preparations; oral antifungals should be avoided [10,14]. Specifically, topical azoles can be used at all stages of pregnancy because there is no or only minimal systemic exposure following intravaginal administration [53]. The FDA assigned topical clotrimazole to pregnancy category B. The other topical imidazoles and triazoles have been assigned to category C. In fact, several clinical trials confirmed the safety of clotrimazole in pregnancy; no association was observed between vaginal application of clotrimazole and congenital abnormalities [54]. 

Our systematic literature search identified five articles which qualified for inclusion (Table 3). In pregnant women with symptomatic vulvovaginal candidosis, clotrimazole 100 mg (vaginal tablet), administered for approximately 1 week, provided high mycological cure rates (78–88% at 1 or 2–4 weeks after therapy). Similar results were observed following 1-week application of clotrimazole 1% internal cream. Intravaginal clotrimazole was significantly more effective than placebo treatment [55]. In the same and other studies, the prophylactic use of clotrimazole during pregnancy significantly lowered the frequency of *Candida* presence on the neonatal skin [55,56,57,58,59]. Moreover, mycological cure rates were markedly higher with multiple-dose clotrimazole compared with nystatin, while there was no difference to terconazole. There was a trend towards reduced effectiveness of clotrimazole when taken as a single intravaginal 500 mg dose. In all five studies, topical clotrimazole was well tolerated.

Two additional randomized controlled trials studied the potential of clotrimazole in the prevention of preterm birth when administered to pregnant women with asymptomatic vaginal candidosis. Kiss et al. [60] evaluated whether a screening strategy in pregnant women presenting for their prenatal visits early in the second trimester lowers the rate of preterm delivery. Subjects with pathological vaginal flora were randomized to an intervention group or control group (no treatment). Women who received intravaginal clotrimazole 100 mg once-daily for 6 days due to asymptomatic vulvovaginal candidosis showed a significantly lower spontaneous preterm birth rate than untreated women (8/294 (2.7%) vs. 22/292 (7.5%); risk ratio (RR): 0.36; 95% confidence interval (CI): 0.16, 0.80) [61]. In a smaller study conducted by Roberts and colleagues [62], 98 pregnant women at <20 weeks of gestation were randomized to intravaginal clotrimazole 100 mg once-daily for 6 days or no treatment. There was a reduction in preterm birth rate in women treated with clotrimazole (4.0% vs. 6.3%), but the difference did not reach statistical significance. A meta-analysis of both trials concluded that treatment of asymptomatic candidosis may reduce the risk of preterm birth, but further prospective, sufficiently powered studies are required [61].

Our findings are in agreement with a previous systematic review on the topical treatment of symptomatic vulvovaginal candidosis in pregnancy. A Cochrane review from 2001 [63] concluded that topical imidazoles are more effective than nystatin and should be used during pregnancy. In addition, short-term treatments with topical imidazole drugs were found to be less effective than 7-day administrations, and topical treatments beyond 7 days provided no additional benefit.

#### 3.2.2. Recurrent Vaginal Yeast Infection

Recurrent symptomatic vaginal yeast infections may have a severe adverse impact on the quality of life. Defined as ≥4 culture-proven episodes per year, it is a long-term condition causing significant morbidity in women [68]. The highest prevalence (9%) has been observed in young women aged 25–34 years [8]. The main fungal species causing recurrent vulvovaginal candidosis is still *Candida albicans*. In fact, in about 85–95% of cases, azole-sensitive *Candida albicans* can be identified as the responsible pathogen. The latter implies that peculiarities of the host (e.g., genetic factors that facilitate vaginal colonization and persistence) must contribute to the development of disease recurrence [68]. Optimized treatment regimens are required to combat this debilitating and complex disease. Usually, an induction course followed by maintenance or intermittent treatment for at least 6 months is pursued [14].

We found four randomized controlled studies investigating intravaginal clotrimazole in non-pregnant women with recurrent symptomatic vulvovaginal candidosis (Table 4). Clotrimazole induction treatment for 1–2 weeks (e.g., clotrimazole 100 mg once-daily) resulted in short-term mycological and clinical cure rates of >80%. Intermittent once-monthly prophylactic administration of clotrimazole 500 mg (solid system) for 6 months prevented clinical disease recurrence in up to 70% of women, but did not prevent vaginal recolonization with *Candida albicans* in the majority of cases. More subjects on intermittent intravaginal clotrimazole remained asymptomatic than subjects receiving intermittent oral itraconazole or placebo. In one head-to-head study, clotrimazole and oral ketoconazole induction treatments were equally effective at achieving high short-term cure rates, but without immediate initiation of maintenance/intermittent therapy, longer-term recurrence rates were high in both groups [69]. Except for occasional vulvovaginal burning, topical clotrimazole was well tolerated in all four studies. Adverse events were significantly more common with oral itraconazole and oral ketoconazole than with intravaginal clotrimazole.

Our findings summarized in Table 4 must be put into perspective with the fact that higher cure rates have been reported with oral fluconazole. In a randomized controlled study in 387 women with recurrent symptomatic vulvovaginal candidosis, oral fluconazole 150 mg was administered on days 0, 3, 6, followed by once-weekly intakes for 6 months. The proportion of women who remained clinically cured until 6 months was 91% in the fluconazole group and 36% in the placebo group (*p* < 0.001). During the maintenance phase, 2.9% of patients in the fluconazole group and 1.2% in the placebo group reported at least one adverse event that led to discontinuation of the study medication [70].

#### 3.2.3. Severe Vaginal Yeast Infection

Severe vulvovaginal candidosis is characterized by extensive vulvar erythema, swelling, excoriation, itching and fissure formation. Standard treatments as used in uncomplicated vulvovaginal candidosis are insufficient in women with severe disease; intensified treatment regimens comprising repeated doses are required [4,10,14]. 

We identified one article qualifying for inclusion in our review. Zhou et al. [74] conducted a prospective, open, randomized (1:1) study in 240 women with severe vulvovaginal candidosis to assess whether two 500 mg doses of clotrimazole vaginal tablet are as effective as two 150 mg doses of oral fluconazole. In each group, the two doses were administered 3 days apart. Study participants were to be not pregnant and at least 18 years old. In 90% of cases, *Candida albicans* was the causative pathogen. The mycological cure rates at days 7–14 after therapy were 78% and 74% (*p* = 0.147) in the clotrimazole group and the fluconazole group, respectively. The corresponding clinical cure rate was 89% in both groups. At days 30–35, the mycological cure rates amounted to 54% and 56% (*p* = 0.813) in the clotrimazole group and the fluconazole group, respectively; the clinical cure rates at that time point were 72% and 78% (*p* = 0.298), respectively. Systemic adverse events were more common with oral fluconazole (e.g., headache) than with intravaginal clotrimazole, while local adverse events (e.g., mild burning) occurred more frequently in the clotrimazole group. It was concluded that the two tested treatment regimens are equally effective, with a safety benefit for clotrimazole.

#### 3.2.4. Vaginal Yeast Infection in Immunocompromised Host

Vulvovaginal candidosis is of particular concern in immunocompromised subjects such as women with poorly controlled diabetes mellitus, HIV-infection, or on immunosuppressive drugs. Usually, these populations require prolonged conventional antifungal therapy for 7–14 days, including intravaginal azole therapy [4,11,14].

We found no qualifying study that investigated the therapeutic effectiveness of intravaginal clotrimazole in this setting. However, Williams and colleagues performed a randomized, double-blind, placebo-controlled study on the potential of intravaginal clotrimazole to prevent vaginal candidosis in adult women with HIV [75]. In the clotrimazole arm, study participants applied the drug (capsules containing clotrimazole powder 100 mg) once a week. At 6-month intervals, vaginal samples were collected and examined. The risk of experiencing an episode of vulvovaginal candidosis during clotrimazole use was significantly reduced compared to placebo (risk ratio: 0.4; 95% CI: 0.2, 0.9].

#### 3.2.5. Non-Albicans Vaginal Yeast Infection

In about 10% of cases, non-*albicans Candida* species are responsible for acute symptomatic vulvovaginal candidosis [14]. Among them, *Candida glabrata* is the most frequently identified strain [68]. Non-*albicans Candida* species as causative pathogens of vulvovaginal candidosis are an emerging threat. Risk factors include uncontrolled type 2 diabetes and advanced age as well as intake of glycosuria-inducing agents to manage type 2 diabetic patients [68]. The optimal treatment of non-*albicans* vaginal yeast infections has not been established [4]. Non-*albicans Candida* infections are unlikely to respond to standard treatments. In fact, azoles are poorly effective in the treatment of *Candida glabrata*-caused vulvovaginal candidosis [10]. Boag and co-workers [76] investigated the effect of oral fluconazole and intravaginal clotrimazole on the vaginal microbial flora. The vaginal flora was unaltered after both therapies. In women suffering from vaginitis due to *Candida glabrata* or *Candida krusei*, the yeasts persisted longer and revealed a poorer treatment response to either treatment.

Our literature search did not identify qualifying clotrimazole-studies in this setting.

### 3.3. Clotrimazole in Men with Candida Balanitis

Balanitis affects approximately 3–11% of men at least once during lifetime. Fungal microorganisms—especially *Candida albicans*—are most frequently responsible for this itching inflammation of the glans penis [5]. *Candida* balanitis is not considered a sexually transmitted disease, but in approximately 20% of male partners of women with recurrent vulvovaginal candidosis, *Candida* species can be isolated on their penises [10]. In one study, 43% of men with *Candida* balanitis had partners with *Candida* vaginitis [77]. In another study, 107 male partners of women with acute vaginal candidosis were examined, and 45% exhibited symptoms of balanitis [48]. Those men who develop *Candida* balanitis benefit from topical antifungal treatment [4].

In a randomized (1:1), open-label study, Stary et al. [77] compared the effectiveness and safety of a single oral dose of fluconazole 150 mg with clotrimazole 1% external cream applied topically twice-daily for 7 days in 157 adult men with balanitis. In those study participants with a positive baseline culture for *Candida albicans*, 49/63 (78%) subjects in the fluconazole group and 53/64 (83%) subjects in the clotrimazole group were mycologically cured at days 8–11 after initiation of therapy (*p* > 0.05). At the same time-point, 92% and 91% (*p* > 0.05), respectively, were clinically cured or had improved. At the one-month follow-up visit, 26/36 (72%) subjects in the fluconazole group and 25/33 (76%) subjects in the clotrimazole group were mycologically cured. The median time to relief of symptoms (erythema) was 6 days during fluconazole treatment and 7 days with clotrimazole. Both treatments were well tolerated. It was concluded that both treatments are equipotent in the treatment of *Candida* balanitis.

Maw et al. [78] conducted an open, comparative study of bifonazole and clotrimazole in adults with candidal balanoposthitis (i.e., both the glans and the foreskin were affected). Study participants were randomized to receive either bifonazole 1% cream once-daily for 6 days or clotrimazole 1% cream twice-daily for 6 days. On day 7 after treatment initiation, 21/27 (78%) subjects in the bifonazole group and 20/26 (77%) subjects in the clotrimazole group were mycologically cured. The difference was not statistically significant. No adverse events were reported in the clotrimazole group while one subject in the bifonazole group reported increased erythema following initial application.

In an uncontrolled study in 138 men with *Candida* balanitis, clotrimazole 1% external cream applied topically twice-daily for 7 days yielded mycological cure rates of 90% at day 7 after the start of therapy. Treatment was acceptable even to subjects who continued to engage in sexual activity during treatment [79].

## 4. Summary

More than 45 years ago, the first topical clotrimazole formulation (Canesten^®^ vaginal tablet) was registered. Today, it is available in different dosages and formats allowing treatments according to individual needs and preferences. Various studies and its therapeutic use over decades confirmed the antimycotic activity of clotrimazole in the treatment of vulvovaginal candidosis. Despite its use over many years, clotrimazole resistance in vaginal candidosis is rare, with the caveat that drug resistance has emerged particularly in immunocompromised patients [1,12]. Resistance has been associated with the overexpression of efflux pump genes. Changes in the clotrimazole target, the fungal cytochrome 14α-demethylase enzyme, may also play a role in some cases [1]. We conducted a systematic literature search on the effectiveness and local tolerability of topical clotrimazole when used for the treatment of uncomplicated and complicated vulvovaginal candidosis, and when used topically by men with *Candida* balanitis. In total, 39 randomized controlled studies qualified for inclusion in our review.

In women with uncomplicated (acute) vulvovaginal candidosis, single topical doses of a clotrimazole 500 mg vaginal tablet provided mycological cure rates of up to 95%. The single use of clotrimazole 10% internal cream or clotrimazole 500 mg ovule resulted in similar results. This is in accordance with the observation that fungicidal concentrations of clotrimazole could be determined in vaginal secretions up to three days after insertion of one vaginal 500 mg tablet [80]. In head-to-head comparative studies, single-dose regimens of intravaginal clotrimazole and oral antifungals were equally effective. In cases when the candidosis extended to the vulvar region, the combined use of intravaginal clotrimazole and external clotrimazole cream provided added benefit (e.g., less local itching). In fact, two observational studies including over 5800 women with vulvovaginal mycosis showed that more than 75% of physicians prefer to treat their patients with a combination of clotrimazole to be applied intravaginally (vaginal tablet or cream) and clotrimazole cream to be applied externally to the vulva and surrounding areas [19,81].

Topical clotrimazole has also proved its effectiveness in the therapy of complicated vulvovaginal candidosis. In pregnant women with symptomatic vaginal yeast infection, one-week treatments with clotrimazole 100 mg or 1% internal cream were associated with high mycological cure rates. Furthermore, the prophylactic use of clotrimazole during pregnancy significantly lowered the risk for *Candida* infections of newborns and preterm birth. 

In recurrent symptomatic vulvovaginal candidosis, clotrimazole induction treatment for 1–2 weeks resulted in short-term mycological cure rates of >80%, and topical treatments applied intermittently (monthly) prevented disease recurrence and breakthrough vaginitis in up to 70%. However, higher clinical cure rates were observed with once-weekly oral fluconazole. In one head-to-head comparative study in women with severe vulvovaginal candidosis, two 500 mg doses of clotrimazole vaginal tablet, administered 3 days apart, were as effective as two 150 mg doses of oral fluconazole, whereas intravaginal clotrimazole was better tolerated. No high-quality studies were found on the usefulness of topical clotrimazole in the therapy of non-*albicans* vaginal yeast infections and in the treatment of vulvovaginal candidosis in immunocompromised hosts. 

Results from two studies provided convincing scientific evidence that clotrimazole 1% external cream, applied topically twice-daily for one week, is associated with high mycological cure rates in men with *Candida* balanitis. In fact, the latter regimen was as effective as a single oral dose of fluconazole 150 mg. 

In all studies, topical clotrimazole was well tolerated; mild local adverse events (e.g., burning) were reported occasionally. 

Based on our findings, principal clotrimazole treatment regimens can be inferred. Those are summarized in Table 5. 

The results of our literature search are also mirrored in several treatment guidelines. In the guideline issued by the U.S. Centers for Disease Control and Prevention (CDC), it reads: “Short-course topical formulations (i.e., single dose and regimens of 1–3 days) effectively treat uncomplicated vulvovaginal candidosis. The topically applied azole drugs are more effective than nystatin.” [4]. In various other treatment guidelines, short course intravaginal clotrimazole (i.e., vaginal tablet 500 mg as a single dose or 200 mg once-daily for three days) belongs to the recommended regimens for treating uncomplicated vulvovaginal candidosis [12,14,74]. In the World Health Organization (WHO) guideline on the management of vaginal discharge, it reads: “The Guidelines Group recommends that the current best treatment for candida in pregnant women are topical azole preparations. Topical azoles can be used at any stage of pregnancy for treatment of symptomatic candidosis” [14]. This is in agreement with other guidelines [4,12]. In terms of recurrent vulvovaginal candidosis, the most frequently recommended regimen consists of an induction course and maintenance therapy with fluconazole (weekly for six months) [13]. However, in cases when this regimen is not feasible, intermittent topical treatments are also suitable options [4,14]. The American College of Obstetricians and Gynecologists suggests a maintenance therapy with topical clotrimazole 500 mg once-weekly if affected women are “unable or unwilling” to take oral fluconazole [13]. Specific induction therapies can be found in the Canadian Guidelines. Among the recommended regimens, there is oral fluconazole 150 mg, once every 72 h for three doses, and a topical azole for 10–14 days. Maintenance therapy may consist of oral fluconazole 150 mg once-weekly or clotrimazole 500 mg intravaginally once-monthly [82]. According to the CDC, severe vulvovaginal candidosis should be treated with a topical azole for 7–14 days or with two oral doses of fluconazole 150 mg (administered three days apart) [4]. Of note, two doses of clotrimazole vaginal tablet 500 mg (administered three days apart) were shown to be as effective as two doses of oral fluconazole 150 mg, suggesting that both regimens represent suitable treatment options in subjects with severe vulvovaginal candidosis [74]. 

For the treatment of balanitis/balanoposthitis, the WHO recommended topical application of clotrimazole twice-daily for 7 days or nystatin [83]. In fact, imidazoles such as topical clotrimazole 1% twice-daily belong to the drugs of choice in the treatment of *Candida* balanitis [5].

## 5. Conclusions

Almost 50 years ago, the first topical clotrimazole formulation was registered for the local treatment of vulvovaginal candidosis. Since then, different formulations have been developed and tested in a variety of studies. Cumulative evidence from numerous randomized controlled trials provided convincing scientific evidence that topical clotrimazole is effective and safe in the therapy of uncomplicated (acute) and certain types of complicated vaginal yeast infections, as well as *Candida* balanitis. The drug still belongs to the first-line treatments in these settings as specified in several treatment guidelines. It is therefore expected that clotrimazole will continue to be widely used in the field of vaginal health in future, more so as clotrimazole resistance in vaginal candidosis occurs rarely, despite its global use over many years.

## Figures and Tables

**Table 1:** Placebo- and active controlled studies investigating intravaginal clotrimazole in women with uncomplicated (acute) vulvovaginal candidosis.

Reference and Population	Design	Fungal Verification Method	*Candida* Species	Drug and Formulation	Regimen	Outcomes	Adverse Events
[26] Adolescents ≥16 y and adults	r, db, pc	Culture and microscopy	*C. albicans*	CLO vaginal tablet or placebo	CLO: 500 mg, single dose, *n* = 10 Placebo: *n* = 13	Mycological cure rate at D 5–31 after Rx (CLO vs. placebo): 90% vs. 0%, *p* = 0.0001 Clinical cure rate at D 5–31 after Rx (CLO vs. placebo): 90% vs. 0%, *p* = 0.0001	None occurred
[27] Adolescents ≥16 y and adults	r, db, pc, mc	Culture	*C. albicans* (65% of subjects)	CLO vaginal tablet or placebo	CLO: 500 mg, single dose, *n* = 55 Placebo: *n* = 40	Mycological cure rate at W 1 after Rx (CLO vs. placebo): 62% vs. 25%, *p* < 0.001	nr
[28] Adults	r, db *, pc, ac	Culture and microscopy	*C. albicans* (98% of subjects)	CLO vaginal tablet or oral itraconazole capsule or oral placebo capsule	CLO: 200 mg/d for 3 d, *n* = 20 Itraconazole: 200 mg/d for 3 d, *n* = 48 Placebo for 3 d, *n* = 22	Mycological cure rate at W 1 after Rx (CLO vs. itraconazole vs. placebo): 95% vs. 73% vs. 32%, *p* < 0.005 for CLO vs. placebo Mycological cure rate at W 4 (CLO vs. itraconazole vs. placebo): 83% vs. 89% vs. 57%, *p* < 0.05 for CLO vs. placebo Clinical cure rate at W 1 after Rx (CLO vs. itraconazole vs. placebo): 65% vs. 73% vs. 45%, *p* < 0.05 for CLO vs. placebo Clinical cure rate at W 4 (CLO vs. itraconazole vs. placebo): 61% vs. 77% vs. 57%, ns for CLO vs. placebo, ns for CLO vs. itraconazole	CLO: 1 subject with AEs Itraconazole: 17 subjects with AEs Placebo: 9 subjects with AEs
[29] Adults	r, o, ac, mc	Culture and microscopy	*C. albicans* (93% of subjects)	CLO vaginal tablet or oral fluconazole capsule	CLO: 200 mg qd for 3 d, *n* = 181 Fluconazole: 150 mg, single dose, *n* = 188	Mycological cure rate at D 5–16 after Rx (CLO vs. fluconazole): 81% vs. 85%, ns Mycological cure rate at D 27–62 (CLO vs. fluconazole): 62% vs. 72%, ns	CLO: 9 subjects with mild AEs Fluconazole: 8 subjects with mild AEs
[30] Adolescents ≥15 y and adults	r, o, ac	Culture and microscopy	*C. albicans*	CLO vaginal tablet or oral fluconazole capsule	CLO: 500 mg, single dose, *n* = 20 Fluconazole: 150 mg, single dose, *n* = 23	Mycological cure rate at D 8 after Rx (CLO vs. fluconazole): 75% vs. 87%, *p* = 0.05 Mycological cure rate at D 32 (CLO vs. fluconazole): 60% vs. 87%, *p* = 0.05	CLO: 2 subjects with mild AEs Fluconazole: 1 subject with mild AEs
[31] Adults	r, o, ac, mc	Culture	nr	CLO vaginal tablet or oral fluconazole capsule	CLO: 200 mg qd for 3 d, *n* = 95 Fluconazole: 50 mg qd for 3 d, *n* = 90	Mycological cure rate at D 7–10 after Rx (CLO vs. fluconazole): 93% vs. 89%, nc Mycological cure rate at D 30–35 (CLO vs. fluconazole): 86% vs. 77%, nc Clinical cure rate at D 7–10 after Rx (CLO vs. fluconazole): 88% vs. 84%, nc Clinical cure rate at D 30–35 (CLO vs. fluconazole): 83% vs. 79%, nc Mycological cure rate at D 6–8 after Rx (CLO vs. fluconazole): 84% vs. 86%, ns	CLO: 5 subjects with mild/moderate AEs Fluconazole: 12 subjects with mild/moderate AEs One subject on fluconazole discontinued due to diarrhea
[32] Adolescents ≥16 y and adults	r, o, ac, mc	Culture and microscopy	*C. albicans* (92% of subjects)	CLO vaginal tablet + CLO external 1% cream or oral fluconazole capsule	CLO: 500 mg, single dose + cream on vulva bid, *n* = 92 Fluconazole: 150 mg, single dose, *n* = 93	Mycological cure rate at D 28–32 (CLO vs. fluconazole): 85% vs. 89%, ns Clinical cure rate at D 6–8 after Rx (CLO vs. fluconazole): 80% vs. 87%, ns Clinical cure rate at D 28–32 (CLO vs. fluconazole): 84% vs. 92%, ns	CLO: 3 subjects with AEs Fluconazole: 2 subjects with AEs
[33] Adults	r, o, ac, mc	Culture	nr	CLO vaginal tablet or oral fluconazole capsule	CLO: 500 mg, single dose Fluconazole: 150 mg, single dose Total *n* = 537	Mycological cure rate at D 7 after Rx (CLO vs. fluconazole): 76% vs. 82%, ns Mycological cure rate at D 28 (CLO vs. fluconazole): 72% vs. 75%, ns	CLO: 110 AEs Fluconazole: 112 AEs Both drugs well accepted
[34]	r, o, ac	Culture and microscopy	nr	CLO vaginal tablet or oral fluconazole	CLO: 100 mg qd for 6 d, *n* = 50 Fluconazole 1: 50 mg qd for 6 d, *n* = 90 Fluconazole 2: 150 mg, single dose, *n* = 50	Mycological cure rate at D 5–15 after Rx (CLO vs. fluconazole 1 vs. fluconazole 2): 72% vs. 88% vs. 76%, nc Mycological cure rate at D 30–60 (CLO vs. fluconazole 1 vs. fluconazole 2): 60% vs. 80% vs. 70%, nc Clinical cure rate at D 5–15 after Rx (CLO vs. fluconazole 1 vs. fluconazole 2): 72% vs. 92% vs. 80%, ns Clinical cure rate at D 30–60 (CLO vs. fluconazole 1 vs. fluconazole 2): 58% vs. 88% vs. 76%, ns	None occurred
[35] Adults	r, sb, ac, mc	Culture	*C. albicans* (85–88% of subjects)	CLO vaginal tablet or oral fluconazole capsule	CLO: 100 mg qd for 7 d, *n* = 214 Fluconazole: 150 mg, single dose, *n* = 218	Mycological cure rate at D 14 after start of Rx (CLO vs. fluconazole): 72% vs. 77%, ns Mycological cure rate at D 35 (CLO vs. fluconazole): 57% vs. 63%, ns Clinical cure rate at D 14 after start of Rx (CLO vs. fluconazole): 67% vs. 73%, ns Clinical cure rate at D 35 (CLO vs. fluconazole): 75% vs. 75%, ns	CLO: 37 subjects with mild/moderate AEs Fluconazole: 59 subjects with mild/moderate AEs
[36] Adults	r, sb, ac	Culture	nr	CLO vaginal tablet or oral fluconazole capsule	CLO: 100 mg bid for 3 d, *n* = 50 Fluconazole: 150 mg, single dose, *n* = 53	Mycological cure rate at W 1 after Rx (CLO vs. fluconazole): 80% vs. 79%, ns Mycological cure rate at W 4 (CLO vs. fluconazole): 66% vs. 60%, ns	CLO: 11 subjects had mild vaginal burning sensations Fluconazole: 4 subjects with mild nausea or dizziness
[37] Adolescents ≥16 y and adults	r, sb, ac	Culture and microscopy	nr	CLO vaginal tablet + CLO external 1% cream or oral fluconazole or oral itraconazole	CLO: 500 mg, single dose, *n* = 82 Fluconazole: 150 mg, single dose, *n* = 72 Itraconazole: 200 mg bid for 1 d, *n* = 75	Mycological cure rate at D 7–10 (CLO vs. fluconazole vs. itraconazole): 95% vs. 83% vs. 96%, *p* = 0.033 for CLO vs. fluconazole Clinical cure rate at D 7–10 (CLO vs. fluconazole vs. itraconazole): 80% vs. 62% vs. 80%, *p* = 0.027 for CLO vs. fluconazole	nr
[38] Adults	r, o, ac	Culture and microscopy	nr	CLO vaginal tablet or oral fluconazole or oral itraconazole	CLO: 100 mg qd for 6 d, *n* = 50 Fluconazole: 150 mg, single dose, *n* = 50 Itraconazole: 200 mg qd for 3 d, *n* = 50	Mycological cure rate at D 5–15 after Rx (CLO vs. fluconazole vs. itraconazole): 72% vs. 76% vs. 80%, nc Mycological cure rate at D 30–60 (CLO vs. fluconazole vs. itraconazole): 60% vs. 70% vs. 74%, nc Clinical cure rate at D 5–15 after Rx (CLO vs. fluconazole vs. itraconazole): 72% vs. 80% vs. 84%, nc Clinical cure rate at D 30–60 (CLO vs. fluconazole vs. itraconazole): 58% vs. 76% vs. 78%, nc	None occurred
[21] nr	r, sb, ac, mc	Culture and microscopy	*C. albicans* (95% of subjects)	CLO vaginal tablet + CLO external 1% cream (VT) or CLO 10% vaginal cream + CLO external 2% cream (VC) or oral fluconazole capsule	CLO VT: 500 mg, single dose, *n* = 226 CLO VC: 1 tube, single dose, *n* = 226 Fluconazole: 150 mg, single dose, *n* = 227	Mycological cure rate at D 14 (CLO VT vs. CLO VC vs. fluconazole): 80% vs. 74% vs. 76%, CLO VT and CLO VC non-inferior to fluconazole Clinical cure rate at D 14 (CLO VT vs. CLO VC vs. fluconazole): 66% vs. 61% vs. 59%, CLO VT and CLO VC non-inferior to fluconazole	CLO VT: 26 subjects with mild AEs CLO VC: 32 subjects with mild AEs Fluconazole: 29 subjects with mild AEs
[39] Adolescents >15 y and adults	r, o, ac	Microscopy	nr	CLO vaginal tablet or oral fluconazole	CLO: 200 mg qd for 6 d, *n* = 70 Fluconazole: 150 mg, single dose, *n* = 72	Mycological cure rate at D 7 after Rx (CLO vs. fluconazole): 70% vs. 83%, *p* = 0.01 Clinical cure rate at D 7 after Rx (CLO vs. fluconazole): 59% vs. 74%, *p* = 0.001	CLO: pelvic pain Fluconazole: headache No difference in AE frequencies
[40] Adolescents ≥16 y and adults	r, sb, ac, mc	Culture	nr	CLO vaginal tablet or oral ketoconazole	CLO: 100 mg qd for 6 d, *n* = 29 Ketoconazole: 200 mg bid for 5 d, *n* = 34	Mycological cure rate at W 2 (CLO vs. ketoconazole): 86% vs. 83%, nc	CLO: 1 subject with AEs Ketoconazole: 5 subjects AEs
[41] Adolescents ≥16 y and adults	r, o, ac	Culture and microscopy	*C. albicans* (96% of subjects)	CLO vaginal ovule or oral ketoconazole or CLO + lactic acid in vaginal ovule	CLO: 500 mg, single dose, *n* = 25 Ketoconazole: 200 mg bid for 5 d, *n*= 25 CLO + lactic acid: 500 mg, single dose, *n* = 25	Mycological cure rate at W 3 after Rx (CLO vs. ketoconazole vs. CLO + lactic acid): 84% vs. 76% vs. 92%, nc	nr
[42] Adults	r, sb, ac, mc	Culture	nr	CLO vaginal tablet or oral itraconazole capsule	CLO: 500 mg, single dose, *n* = 105 Itraconazole: 200 mg bid for 1 d, *n* = 109	Mycological cure rate at W 1 after Rx (CLO vs. itraconazole): 72% vs. 74%, ns Mycological cure rate at W 6 (CLO vs. itraconazole): 50% vs. 51%, ns	nr

ac: active-controlled; AEs: adverse events; bid: twice-daily; CLO: clotrimazole; d or D: day; db: double-blind; M: month; mc: multicenter; *n*: number of subjects; nc: not compared; nr: not reported; ns: not statistically significant; o: open; pc: placebo-controlled; qd: once-daily; r: randomized; Rx: treatment; sb: single-blind; W: week; y: year. * Comparison with clotrimazole was not blinded.

**Table 2:** Randomized controlled studies comparing different treatment regimens or formulations of intravaginal clotrimazole in women with uncomplicated (acute) vulvovaginal candidosis.

Reference and Population	Design	Fungal Verification Method	*Candida* Species	Formulations	Regimen	Outcomes	Adverse Events
[43] Adults	r, db, ac	Culture	*C. albicans*	CLO vaginal tablet 500 mg or CLO vaginal tablet 200 mg	CLO 1: 500 mg, single dose, *n* = 35 CLO 2: 200 mg qd for 3 d, *n* = 37	Mycological cure rate at D 7 after Rx (CLO 1 vs. CLO 2): 94% vs. 89%, ns Clinical cure rate at W 4 after Rx (CLO 1 vs. CLO 2): 86% vs. 92%, ns	None occurred
[44] Adults	r, db, ac, mc	Culture and microscopy	nr	CLO vaginal tablet 500 mg or CLO vaginal tablet 100 mg	CLO 1: 500 mg, single dose, *n* = 53 CLO 2: 2 × 100 mg qd for 3 d, *n* = 50	Mycological + clinical cure rate at D 5–10 after Rx (CLO 1 vs. CLO 2): 90% vs. 89%, ns Mycological + clinical cure rate at ≥D 27 (CLO 1 vs. CLO 2): 75% vs. 72%, ns	CLO 2: 1 subject had moderate edema of vulva
[45] Adults	r, db, ac	Culture and microscopy	nr	CLO vaginal tablet 500 mg or CLO vaginal tablet 100 mg	CLO 1: 500 mg, single dose, *n* = 18 CLO 2: 2 × 100 mg qd for 3 d, *n* = 18	Mycological + clinical cure rate at M 1 after Rx (CLO 1 vs. CLO 2): 89% vs. 83%, ns	CLO 2: 1 subject had moderate edema of vulva
[46] Adults	r, db, ac	Culture and microscopy	*C. albicans*	CLO vaginal tablet 500 mg or CLO vaginal tablet 100 mg	CLO 1: 500 mg, single dose, *n* = 14 CLO 2: 2 × 100 mg qd for 3 d, *n* = 13	Mycological + clinical cure rate at M 1 after Rx (CLO 1 vs. CLO 2): 86% vs. 85%, ns	None occurred
[47] Adults	r, db, ac	Culture and microscopy	nr	CLO vaginal tablet 500 mg or CLO vaginal tablet 100 mg	CLO 1: 500 mg, single dose, *n* = 20 CLO 2: 100 mg qd for 6 d, *n* = 20	Mycological cure rate at D 3 after Rx (CLO 1 vs. CLO 2): 85% vs. 95%, nc Mycological cure rate at W 4 after Rx (CLO 1 vs. CLO 2): 95% vs. 80%, nc	1 subject reported vaginal burning
[21] nr	r, sb, ac, mc	Culture and microscopy	*C. albicans* (95% of subjects)	CLO vaginal tablet 500 mg + CLO external 1% cream (VT) or CLO 10% vaginal cream + CLO external 2% cream (VC) or oral fluconazole capsule	CLO VT: 500 mg, single dose, *n* = 226 CLO VC: 1 tube, single dose, *n* = 226 Fluconazole: 150 mg, single dose, *n* = 227	Mycological cure rate at D 14 (CLO VT vs. CLO VC vs. fluconazole): 80% vs. 74% vs. 76%, CLO VT and CLO VC non-inferior to fluconazole Clinical cure rate at D 14 (CLO VT vs. CLO VC vs. fluconazole): 66% vs. 61% vs. 59%, CLO VT and CLO VC non-inferior to fluconazole	CLO VT: 26 subjects with mild AEs CLO VC: 32 subjects with mild AEs Fluconazole: 29 subjects with mild AEs
[48] Adolescents ≥16 y and adults	r, o, ac	Culture	*C. albicans*	CLO 10% cream or CLO 2% cream	CLO 10%: 5 g, single dose, *n* = 55 CLO 2%: 5 g, bid for 3 d, *n* = 55	Mycological cure rate at D 7 after start of Rx (CLO 10% vs. CLO 2%): 91% vs. 96%, nc Mycological cure rate at D 35 (CLO 10% vs. CLO 2%): 84% vs. 81%, nc	None occurred
[49] Adolescents ≥14 y and adults	r, o, ac	Culture and microscopy	*C. albicans* (96% of subjects)	CLO 10% cream or CLO vaginal tablet 500 mg (VT)	CLO 10%: 5 g, single dose, *n* = 81 * CLO VT: 500 mg, single dose, *n* = 82 ^#^	Mycological cure rate at W 1 after Rx (CLO 10% vs. CLO VT): 85% vs. 87%, ns Mycological cure rate at W 4 after Rx (CLO 10% vs. CLO VT): 73% vs. 80%, nc Clinical cure rate at W 1 after Rx (CLO 10% vs. CLO VT): 94% vs. 91%, ns Clinical cure rate at W 4 after Rx (CLO 10% vs. CLO VT): 85% vs. 94%, nc	CLO 10%: 3% of subjects had mild AEs
[22] Adults	r, db, pc, mc	Culture and microscopy	nr	CLO vaginal suppository 200 mg + CLO external 2% cream (T1) or CLO vaginal suppository 200 mg + placebo external cream (T2)	CLO 1 (T1): 200 mg, qd for 3 d, *n* = 79 CLO 2 (T2): 200 mg qd for 3 d, *n* = 79 Cream was applied to the vulva	Mycological cure rate (vagina) at D 6 after Rx (CLO 1 vs. CLO 2): 92% vs. 90%, ns Mycological cure rate (vulva) at D 6 after Rx (CLO 1 vs. CLO 2): 73% vs. 52%, *p* = 0.005 Mycological cure rate (vagina) at W 4 (CLO 1 vs. CLO 2): 23/25 (92%) vs. 21/23 (91%), ns Mycological cure rate (vulva) at W 4 (CLO 1 vs. CLO 2): 16/23 (70%) vs. 13/21 (62%), ns	CLO 1: 1 subject reported vaginal burning CLO 2: 1 subject reported skin peeling With CLO 1, significantly less local itching and extravaginal redness
[50] Adolescents ≥14 y and adults	r, sb, ac, mc	Culture and microscopy	nr	CLO ovule 500 mg (O) or CLO vaginal tablet 500 mg (VT)	CLO 1 (O): 500 mg, single dose, *n* = 237 CLO 2 (VT): 500 mg, single dose, *n* = 228	Mycological cure rate at W 2 after Rx (CLO 1 vs. CLO 2): 81% vs. 78%, ns Mycological cure rate at W 6–8 (CLO 1 vs. CLO 2): 78% vs. 81%, ns Clinical cure rate at W 2 after Rx (CLO 1 vs. CLO 2): 88% vs. 84%, ns Clinical cure rate at W 6–8 (CLO 1 vs. CLO 2): 93% vs. 93%, ns	CLO 1: 1 subject reported a mild drug-related AE (vulvovaginal discomfort)

ac: active-controlled; AEs: adverse events; bid: twice-daily; CLO: clotrimazole; d or D: day; db: double-blind; mc: multicenter; *n*: number of subjects; nc: not compared; nr: not reported; ns: not statistically significant; o: open; pc: placebo-controlled; qd: once-daily; r: randomized; Rx: treatment; sb: single-blind; W: week. * Seven women were pregnant; ^#^ One woman was pregnant.

**Table 3:** Randomized controlled studies investigating intravaginal clotrimazole in pregnant women with symptomatic vulvovaginal candidosis.

Reference	Design	Fungal Verification Method	*Candida* Species	Drug and Formulation	Regimen	Outcomes	Adverse Events
[55]	r, db, pc	Culture	*C. albicans*	CLO vaginal tablets 100 mg + CLO external cream or placebo	CLO: 100 mg + cream qd for 6 d, *n* = 50 Placebo: *n* = 50	Mycological cure rate at W 2–4 after Rx (CLO vs. placebo): 88% vs. 42%, *p* < 0.05	nr
[64]	r, sb, ac	Culture and microscopy	*C. albicans* (90% of subjects)	CLO vaginal tablet 100 mg or nystatin vaginal tablet 100,000 IU	CLO: 100 mg qd for 6 d, *n* = 33 Nystatin: 200,000 IU qd for 6 d, *n* = 29	Mycological cure rate at W 1 after Rx (CLO vs. nystatin): 78% vs. 41%, *p* < 0.01 Mycological cure rate at W 5 after Rx (CLO vs. nystatin): 21/23 (91%) vs. 7/23 (32%), *p* < 0.005	None occurred
[65]	r, o, ac	Culture and microscopy	nr	CLO vaginal tablet 100 mg or nystatin vaginal tablet 100,000 IU	CLO: 100 mg qd for 11 d, *n* = 21 Nystatin: 200,000 IU qd for 11 d, *n* = 19	Mycological cure rate at D 3 after Rx (CLO vs. nystatin): 76% vs. 63%, ns Mycological cure rate at W 6 after Rx (CLO vs. nystatin): 5/7 (71%) vs. 1/7 (14%), ns	None occurred
[66]	r, db, ac	Culture and microscopy	*C. albicans* (99% of subjects)	CLO 1% cream or terconazole 0.4% cream	CLO: 5 g qd for 7 d, *n* = 19 Terconazole: 5 g qd for 7 d, *n* = 19	Mycological + clinical cure rate at W 1 after Rx (CLO vs. terconazole): 95% vs. 95%, ns Mycological + clinical cure rate at W 4 after Rx (CLO vs. terconazole): 84% vs. 79%, ns	Terconazole: 1 subject reported mild vaginal burning
[67]	r, db, ac	Culture and microscopy	nr	CLO vaginal tablet 500 mg or CLO vaginal tablet 100 mg	CLO 1: 500 mg, single dose, *n* = 48 * CLO 2: 2 × 100 mg qd for 3 d, *n* = 53 ^#^	Mycological cure rate at ≥W 4 after Rx (CLO 1 vs. CLO 2): 67% vs. 75%, ns	CLO 1: 1 subject reported vulvar lesion CLO 2: 2 subjects with AEs (rash, vulvar lesion)

ac: active-controlled; AEs: adverse events; CLO: clotrimazole; d or D: day; db: double-blind; IU: international unit; *n*: number of subjects; nr: not reported; ns: not statistically significant; o: open; pc: placebo-controlled; qd: once-daily; r: randomized; Rx: treatment; sb: single-blind; W: week. * Four women were not pregnant; ^#^ Five women were not pregnant.

**Table 4:** Randomized controlled studies investigating intravaginal clotrimazole in women with recurrent symptomatic vulvovaginal candidosis.

Reference and Population	Design	Fungal Verification Method	*Candida* Species	Drug and Formulation	Regimen	Outcomes	Adverse Events
[71] Adults	r, db, pc	Culture and microscopy	*C. albicans* (86% of subjects)	CLO vaginal suppository or placebo	Phase 1 (Rx) (all subjects) CLO: 500 mg qw for 2 w, *n* = 42 Phase 2 (Pro) * CLO: 500 mg qm for 6 m, *n* = 15 Placebo: *n* = 12	Phase 1 Mycological cure rate at W 2 after Rx: 83% Clinical cure rate at W 2 after Rx: 90% Phase 2 Mycologically still cured at M 6 (CLO vs. placebo): ~20% ^#^ vs. ~5% ^#^, ns Clinically still cured at M 6 (CLO vs. placebo): 47% vs. 33%, ns	None occurred
[72] Adults	r, db, pc	Culture and microscopy	*C. albicans*	CLO vaginal tablet or placebo	Phase 1 (Rx) (all subjects) CLO: 500 mg, single dose Phase 2 (Pro) ^$^ CLO: 500 mg qm for 6 m, *n* = 33 Placebo: *n* = 29	Phase 1 Mycological and clinical cure rates: nr Phase 2 Mycologically still cured at M 6 (CLO vs. placebo): 30% vs. 14%, ns Clinically still cured at M 6 (CLO vs. placebo): 70% vs. 21%, *p* < 0.001	None occurred
[73] Adults	r, o, ac	Culture and microscopy	*C. albicans*	CLO vaginal ovules or oral itraconazole	CLO: 200 mg qd for 5 d, then 200 mg biw for 6 m, *n* = 22 Itraconazole: 100 mg bid for 5 d, then 200 mg biw for 6 m, *n* = 22	Mycological + clinical cure rate at M 6 after Rx (CLO vs. itraconazole): 17/17 (100%) vs. 14/21 (67%), *p* = 0.02	Itraconazole: 32% of subjects had mild AEs
[69] Adults	r, o, mc	Culture and microscopy	nr	CLO vaginal suppository or oral ketoconazole	CLO: 100 mg qd for 7 d, *n* = 77 Ketoconazole: 400 mg qd for 14 d, *n* = 74 No maintenance treatment in both groups	Mycological cure rate at W 1 after Rx (CLO vs. ketoconazole): 82% vs. 80%, ns Clinical cure rate at W 1 after Rx (CLO vs. ketoconazole): 82% vs. 86%, ns Clinical cure rate at M 2 after Rx (CLO vs. ketoconazole): 37% vs. 48%, ns	AEs occurred significantly more frequently with ketoconazole CLO: 3.1% had mild vulvovaginal burning

ac: active-controlled; AEs: adverse events; bid: twice-daily; biw: twice-weekly; CLO: clotrimazole; d or D: day; db: double-blind; mc: multicenter; m or M: month; *n*: number of subjects; nr: not reported; ns: not statistically significant; o: open; pc: placebo-controlled; Pro: prophylaxis; qd: once-daily; qw: once-weekly; qm: once-monthly; r: randomized; Rx: treatment; w or W: week. * Only asymptomatic women, culture status was ignored; ^#^ Estimated from graphical display; ^$^ Only asymptomatic women with negative microscopy and culture.

**Table 5:** Overview of suggested clotrimazole treatment regimens.

Uncomplicated VVC	Complicated VVC			Other Population
≥16 Years of Age	VVC in Pregnancy	Recurrent VVC	Severe VVC	Balanitis
Clotrimazole 500 mg vaginal tablet or vaginal soft ovule as single dose for 1 day or Clotrimazole 10% vaginal cream as single dose for 1 day	Clotrimazole 100 mg vaginal tablet once-daily for 7 days	Induction: Clotrimazole 100 mg vaginal tablet once-daily for 1–2 weeks Maintenance: Clotrimazole 500 mg vaginal tablet or vaginal soft ovule once-monthly for 6 months	Clotrimazole 500 mg vaginal tablet or vaginal soft ovule in two doses 3 days apart	Clotrimazole 1% cream twice-daily for 7 days

VVC: vulvovaginal candidosis.
